# Impact of Neoadjuvant Chemotherapy on the Outcomes of Cytoreductive Surgery and Hyperthermic Intraperitoneal Chemotherapy for Colorectal Peritoneal Metastases: A Multi-Institutional Retrospective Review

**DOI:** 10.3390/jcm9030748

**Published:** 2020-03-10

**Authors:** Eliza W. Beal, Lorena P. Suarez-Kelly, Charles W. Kimbrough, Fabian M. Johnston, Jonathan Greer, Daniel E. Abbott, Courtney Pokrzywa, Mustafa Raoof, Byrne Lee, Travis E. Grotz, Jennifer L. Leiting, Keith Fournier, Andrew J. Lee, Sean P. Dineen, Benjamin Powers, Jula Veerapong, Joel M. Baumgartner, Callisia Clarke, Harveshp Mogal, Marti C. Russell, Mohammed Y. Zaidi, Sameer H. Patel, Vikrom Dhar, Laura Lambert, Ryan J. Hendrix, John Hays, Sherif Abdel-Misih, Jordan M. Cloyd

**Affiliations:** 1 Department of Surgery, Division of Surgical Oncology, The Ohio State University Wexner Medical Center and James Cancer Hospital, Columbus, OH 43201, USA; Eliza.Beal@osumc.edu (E.W.B.); Lorena.Suarez-Kelly@prevea.com (L.P.S.-K.); Charles.Kimbrough@bswhealth.org (C.W.K.); John.Hays@osumc.edu (J.H.); Sherif.Abdel-Misih@stonybrookmedicine.edu (S.A.-M.); 2 Department of Surgery, Division of Surgical Oncology, Johns Hopkins University, Baltimore, MD 21205, USA; fjohnst4@jhmi.edu (F.M.J.); jgreer13@jhmi.edu (J.G.); 3 Department of Surgery, Division of Surgical Oncology, University of Wisconsin, Madison, WI 53792, USA; abbott@surgery.wisc.edu (D.E.A.); cpokrzywa@wisc.edu (C.P.); 4 Department of Surgery, Division of Surgical Oncology, City of Hope National Medical Center, Duarte, CA 91010, USA; mraoof@coh.org (M.R.); bylee@coh.org (B.L.); 5 Department of Surgery, Division of Hepatobiliary and Pancreas Surgery, Mayo Clinic, Rochester, MN 55905, USA; grotz.travis@mayo.edu (T.E.G.); leiting.jennifer@mayo.edu (J.L.L.); 6 Department of Surgical Oncology, University of Texas MD Anderson Cancer Center, Houston, TX 44907, USA; kffourni@mdanderson.org (K.F.); andyjoonlee@gmail.com (A.J.L.); 7 Department of Gastrointestinal Surgery, Moffitt Cancer Center, Tampa, FL 33612, USA; Sean.Dineen@moffitt.org (S.P.D.); Benjamin.Powers@moffitt.org (B.P.); 8 Department of Surgery, Division of Surgical Oncology, University of California San Diego, San Diego, CA 92093, USA; jveerapong@ucsd.edu (J.V.); j1baumgartner@ucsd.edu (J.M.B.); 9 Department of Surgery, Division of Surgical Oncology, Medical College of Wisconsin, Milkwaukee, WI 53226, USA; cnclarke@mcw.edu (C.C.); hmogal@mcw.edu (H.M.); 10 Winship Cancer Institute, Division of Surgical Oncology, Emory University, Atlanta, GA 30322, USA; mrussell@emory.edu (M.C.R.); mzaidi@emory.edu (M.Y.Z.); 11 Department of Surgery, University of Cincinnati, Cincinnati, OH 45219, USA; patel5se@ucmail.uc.edu (S.H.P.); dharvk@ucmail.uc.edu (V.D.); 12 Department of Surgery, Division of Surgical Oncology, University of Massachusetts Memorial Medical Center, Worcester, MA 45219, USA; laura.lambert@hci.utah.edu (L.L.); ryan.hendrix@umassmemorial.org (R.J.H.)

**Keywords:** cytoreductive surgery, hyperthermic intraperitoneal chemotherapy, colorectal peritoneal metastases

## Abstract

Cytoreductive surgery (CRS) with or without hyperthermic intraperitoneal chemotherapy (HIPEC) is associated with improved survival for patients with colorectal peritoneal metastases (CR-PM). However, the role of neoadjuvant chemotherapy (NAC) prior to CRS-HIPEC is poorly understood. A retrospective review of adult patients with CR-PM who underwent CRS+/-HIPEC from 2000–2017 was performed. Among 298 patients who underwent CRS+/-HIPEC, 196 (65.8%) received NAC while 102 (34.2%) underwent surgery first (SF). Patients who received NAC had lower peritoneal cancer index score (12.1 + 7.9 vs. 14.3 + 8.5, *p* = 0.034). There was no significant difference in grade III/IV complications (22.4% vs. 16.7%, *p* = 0.650), readmission (32.3% vs. 23.5%, *p* = 0.114), or 30-day mortality (1.5% vs. 2.9%, *p* = 0.411) between groups. NAC patients experienced longer overall survival (OS) (median 32.7 vs. 22.0 months, *p* = 0.044) but similar recurrence-free survival (RFS) (median 13.8 vs. 13.0 months, *p* = 0.456). After controlling for confounding factors, NAC was not independently associated with improved OS (OR 0.80) or RFS (OR 1.04). Among patients who underwent CRS+/-HIPEC for CR-PM, the use of NAC was associated with improved OS that did not persist on multivariable analysis. However, NAC prior to CRS+/-HIPEC was a safe and feasible strategy for CR-PM, which may aid in the appropriate selection of patients for aggressive cytoreductive surgery.

## 1. Introduction

There are over 1.8 million cases of colorectal cancer (CRC) diagnosed annually and 881,000 annual deaths from CRC, ranking it third in incidence and second in mortality worldwide [1]. Approximately 17% of patients with metastatic CRC have peritoneal metastases (CR-PM), while 2% have the peritoneum as the only site of metastatic disease [2,3]. It has long been known that peritoneal involvement by CRC is a poor prognostic factor as these patients have shorter progression-free and overall survival (OS) compared to patients with other types of metastatic CRC [4,5,6]. In a recent meta-analysis of 14 randomized controlled trials including 10,553 patients with metastatic CRC, OS was better among patients with isolated nonperitoneal sites of metastases than in those with isolated CR-PM [3]. Among patients with several sites of metastases, peritoneal involvement was an independently poor prognostic feature [3].

Historically, CR-PM was considered a terminal condition, associated with a short survival duration and poor quality of life, and was treated with palliative chemotherapy alone. However, as is observed for patients with isolated liver or pulmonary metastases, surgical resection of isolated CR-PM has been found to be associated with survival benefits in appropriately selected patients. Indeed, multiple retrospective series and a few randomized controlled trials have found that cytoreductive surgery (CRS) with or without hyperthermic intraperitoneal chemotherapy (HIPEC) is associated with good long-term survival rates among well-selected patients with CR-PM [2,7,8,9,10,11,12,13,14].

The role of neoadjuvant chemotherapy (NAC) prior to surgery for resectable CR-PM is currently debated, and previous literature on the subject is mixed [7,15,16,17]. Proponents of NAC argue that NAC offers early systemic treatment and improves selection of patients for surgery [18]. On the other hand, NAC may lead to worsened patient physical condition or result in a missed window for surgery [19]. These considerations are similar to the current discussion surrounding the treatment of isolated colorectal liver metastases [20,21]. Furthermore, current guidelines suggest that the use of NAC or proceeding straight to surgery are acceptable alternatives [2]. Given this controversy, we set out to explore the impact of NAC on the short- and long-term outcomes of patients with isolated CR-PM using a multi-institutional dataset.

## 2. Experimental Section

A retrospective review of adult patients with colorectal peritoneal metastases (CR-PM) who underwent cytoreductive surgery (CRS) with or without hyperthermic intraperitoneal chemotherapy (HIPEC) at 12 U.S. institutions from 2000–2017 was performed using the United States Hyperthermic Intraperitoneal Chemotherapy (US HIPEC) Collaborative. The database included all patients treated with CRS ± HIPEC between 1 January 2000 and 1 September 2017. Data were collected at each institution using a standardized data collection sheet. Patients were included in the study if their primary site of disease was adenocarcinoma of the colon or rectum. Patients with extraperitoneal disease were excluded as were those with primary CRC and a peritoneal carcinomatosis index (PCI) score of zero (i.e., prophylactic HIPEC). Institutional review board approval was obtained at the primary, and each additional, site prior to the initiation of data collection.

Preoperative demographic and clinical information was retrospectively recorded for each patient who underwent CRS ± HIPEC. For the purpose of this study, NAC was defined as systemic chemotherapy administered to patients with a diagnosis of CR-PM who subsequently underwent CRS ± HIPEC. Patients who received systemic therapy with neoadjuvant intent but ultimately did not undergo surgery were not included. Intraoperative data recorded included procedures performed, operative time, intraoperative fluid administration, estimated blood loss, PCI score, and completeness of cytoreduction (CC) score. The PCI score grades the extent of peritoneal dissemination on a continuous score ranging from 0–39. The CC score is used to define the completeness of cytoreduction where CC-0 indicates no visible disease remaining, CC-1 indicates nodules less than 2.5 mm persisting after cytoreduction, CC-2 indicates nodules between 2.5 mm and 5.0 cm, and CC-3 suggests nodules greater than 5 cm [22]. Postoperative outcomes including complications, length of stay, and readmissions were recorded. Complications were classified according to the Clavien–Dindo system. Grade I complications include any minor deviation from the planned postoperative course. Grade II complications require pharmacologic management. Grade III complications require interventions performed under local or general anesthesia. Grade IV complications include those that threaten patient life with single- or multiorgan failure. Grade V complications are those that result in patient death. [23]. Long-term outcomes, including overall survival (OS) and recurrence-free survival (RFS), were recorded. OS was defined as time from the date of surgery until death or last follow-up. RFS was defined as time from the date of surgery until clinical or radiographic evidence of recurrence or disease progression on last follow-up.

Patients were divided into two groups—those who received neoadjuvant chemotherapy followed by surgery (NAC group) and those who underwent surgery first (SF). Demographic and clinical characteristics were directly compared between the two groups. Differences between groups were assessed using chi-square tests for categorical variables and Kruskal–Wallis one-way analysis of variance for continuous variables. The short- and long-term outcomes of patients in the NAC group and SF group were directly compared. Kaplan–Meier and univariate and multivariable Cox regression analysis were used to analyze RFS and OS. Factors with *p*-value < 0.1 on univariate analysis as well as the receipt of NAC were included in multivariable analysis.

Two sets of supplemental analyses were performed. The first compared patients who received NAC with bevacizumab versus those who underwent SF. The second examined patients with high-grade tumors (poorly differentiated and/or signet ring cell histologies) who received NAC compared to those with high-grade tumors who underwent SF. For each, Kaplan–Meier and univariate and multivariable Cox regression analysis were used to analyze OS and RFS. For all analyses, STATA 14.2 MP (College Station, TX) was used, and statistical significance set as *p* < 0.05.

## 3. Results

Among 298 patients with isolated CR-PM who met inclusion criteria and underwent CRS ± HIPEC, 196 (66%) received NAC while 102 (34%) underwent SF. Patient demographics and perioperative characteristics are reported in Table 1. In the entire cohort, the mean age was 54.1 years and 54% were female. The majority of patients were American Society of Anesthesiology (ASA) Class III (223, 81%) with an Eastern Cooperative Oncology Group (ECOG) Performance Status score of 0 (140, 59%). There were no significant differences between the two groups in regards to age, gender, ASA class, performance status, most comorbidities, tobacco use, disease-related symptoms, or synchronous peritoneal disease. In the NAC group, 31% of patients had poor tumor differentiation and/or signet ring cells, compared to 17% in the SF group (*p* = 0.087). The chemotherapy regimens used in this cohort varied considerably but were all commonly used regimens for colorectal cancer (Table 2). Fifty-four percent of patients who underwent NAC received bevacizumab.

Patients who received NAC had a lower mean PCI score (12.1 + 7.9 vs. 14.3 + 8.5, *p* = 0.034) and shorter operative time (7.7 + 2.9 vs. 8.7 + 2.7 h, *p* = 0.013), but no difference in rates of CC-0 (74% vs. 66%, *p* = 0.14) compared to the SF group (Table 3). There were no significant differences in rates of 30-day overall complications (61% vs. 60%, *p* = 0.672), grade III/IV complications (22% vs. 17%, *p* = 0.650), hospital readmissions (32% vs. 24%, *p* = 0.114), or 30-day mortality (2% vs. 3%, *p* = 0.411) among patients in the NAC and SF groups, respectively.

**Figure 1:**
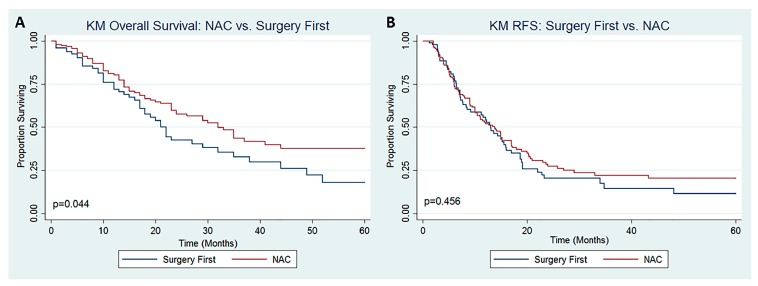
Kaplan–Meier Survival Analysis for (**A**) Overall Survival and (**B**) Recurrence-Free Survival among patients with CR-PM undergoing CRS ± HIPEC.

Patients who received NAC prior to CRS +/- HIPEC experienced a longer OS (Figure 1A, median 32.7 vs. 22.0 months, *p* = 0.044) but similar RFS (Figure 1B, median 13.8 vs. 13.0 months, *p* = 0.456) compared to those who underwent SF. The results of univariate and multivariable cox regression analysis for OS and RFS are reported in Table 4 and Table 5, respectively. After controlling for confounding factors, NAC was not independently associated with improved OS (OR 0.80, 95% CI 0.54–1.17) or RFS (OR 1.04, 95% CI 0.74–1.47). On multivariable Cox regression analysis, increasing PCI score (OR 1.05, 95% CI 1.03–1.08), incomplete cytoreduction (CC-1: OR 2.18, 95% CI 1.31–3.63; CC-2/3: OR 2.37, 95% CI 1.21–4.67), and synchronous CR-PM (OR 0.63, 95% CI 0.42–0.94) were significantly associated with OS (Table 4). Only increasing PCI score (OR 1.05, 95% CI 1.02–1.07) was found to be independently associated with RFS (Table 5).

**Figure 2:**
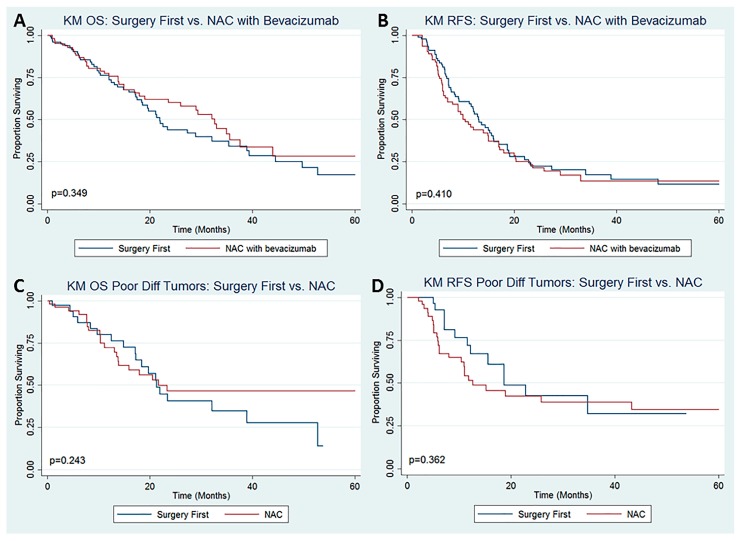
Kaplan–Meier Survival Analysis for (**A**) Overall Survival and (**B**) Recurrence-Free Survival among patients with CR-PM undergoing CRS ± HIPEC comparing patients who underwent NAC with bevacizumab versus those who did not undergo NAC and Kaplan-Meier Survival Analysis for (**C**) Overall Survival and (**D**) Recurrence-Free Survival among patients with CR-PM undergoing CRS ± HIPEC with poorly differentiated tumors ± signet ring histology comparing patients who underwent NAC versus those who underwent surgery first.

On subset analysis comparing patients who underwent NAC with bevacizumab (*n* = 105, 51%) to those who underwent SF (*n* = 102, 49%), there was no difference in the proportion of patients who experienced any perioperative complication (*p* = 0.912). Receipt of NAC + bevacizumab was not a significant predictor of OS (Figure 2A, *p* = 0.349) or RFS (Figure 2B, *p* = 0.410) on Kaplan–Meier analysis. Additionally, receipt of NAC + bevacizumab was not a significant predictor of OS (*p* = 0.449) or RFS (*p* = 0.153) on multivariable Cox proportional hazard models (data not shown). On subset analysis of patients with high-grade tumors (poorly differentiated and/or signet ring cells) comparing those who received NAC (56, 61%) to those who underwent SF (36, 39%), receipt of NAC was not a significant predictor of OS (Figure 2C, *p* = 0.243) or RFS (Figure 2D, *p* = 0.362) on Kaplan–Meier analysis. Receipt of NAC was also not a significant predictor of OS (*p* = 0.084) or RFS (*p* = 0.480) on multivariable Cox proportional hazard models (data not shown). This section may be divided by subheadings. It should provide a concise and precise description of the experimental results, their interpretation as well as the experimental conclusions that can be drawn.

## 4. Discussion

The current study is a large, multi-institutional, retrospective analysis of patients who underwent CRS ± HIPEC. This study was conducted to evaluate the impact of NAC on the short- and long-term outcomes of patients with isolated CR-PM. While patients who received NAC experienced a longer OS compared to those who underwent SF, NAC was not independently associated with improved RFS or OS after controlling for confounding factors. Nevertheless, NAC prior to CRS ± HIPEC appears to be safe and may assist with appropriate selection of patients for aggressive cytoreductive procedures.

The optimal multidisciplinary management of CR-PM continues to be debated [2]. Historically, peritoneal carcinomatosis from metastatic CRC was considered a terminal condition, treated only with palliative chemotherapy. While most patients with CR-PM are still treated with systemic chemotherapy alone, this approach has limited effectiveness due to pharmacokinetic limitations, poor peritoneal penetration, and impaired local drug distribution [4,5,6,24]. CRS-HIPEC has emerged as a locoregional therapy that is associated with improved survival outcomes among well-selected patients treated at experienced centers [7,8,9,10,11,12,13,14]. The recent PRODIGE 7 trial demonstrated impressive median OS of 41 to 42 months in patients treated with CRS +/- HIPEC [25]. Nevertheless, given the challenges in patient selection and the high locoregional and distant recurrence rates following successful surgery, the role of NAC prior to CRS ± HIPEC remains to be defined [15,16,17].

For patients with CR-PM, NAC may be recommended to prioritize systemic treatment and to optimize patient selection for an aggressive loco-regional operation. This approach aims to avoid CRS-HIPEC in patients with rapid disease progression during NAC and improve survival following completion of NAC and CRS-HIPEC. Nevertheless, prior literature on the use of NAC for CR-PM is mixed. Devilee et al., in a retrospective study examining patients with synchronous CR-PM undergoing CRS + HIPEC, reported that receipt of NAC was independently associated with improved OS [26]. In a retrospective review of patients with CR-PM, Passot et al. demonstrated an improvement in OS with NAC on univariate analysis that did not persist on multivariable analysis, similar to the current study [16]. Ceelen et al. demonstrated improvement in OS only when bevacizumab was included in the NAC regimen, a finding that was not replicated in our study [18]. On the other hand, two studies reporting on an overlapping data set demonstrated that the use of NAC was associated with decreased OS [17,27]. Waite et al. performed a systematic review and meta-analysis designed to assess the effect of neoadjuvant and adjuvant chemotherapy on the OS of patients undergoing CRS-HIPEC. Based on seven studies that met inclusion criteria, they concluded that the role of NAC could not be determined by the current evidence [15]. A multicenter Dutch randomized controlled trial is currently underway to test the role of NAC prior to CRS-HIPEC for CR-PM [28].

In addition to its importance for patient selection and early systemic treatment, NAC may be recommended for patients with significant disease burden with the intent of downstaging their disease and improving the odds of complete cytoreduction. Given that incomplete cytoreduction does not improve patient survival, the use of neoadjuvant strategies to increase the odds of obtaining CC0 is naturally appealing. However, the ability of NAC to downstage CR-PM prior to CRS-HIPEC is not well understood [7,11,26,29,30]. The current study demonstrates that patients who received NAC prior to CRS ± HIPEC for CR-PM had lower operative PCI scores compared to those who underwent SF. Whether this is an effect of the NAC or represents biological or selection differences between the two groups is impossible to know via the current study methodology. Therefore, the ability of NAC to downstage disease burden and facilitate complete cytoreduction warrants further investigation via prospective trials.

Although the role of NAC in optimizing long-term outcomes of CRS ± HIPEC remains controversial, the current study suggests its relative safety. While the current study was unable to capture toxicities experienced during NAC itself, both groups of patients had similar performance status and preoperative albumin levels, suggesting that NAC did not significantly lead to physical deconditioning of patients at the time of surgery. Furthermore, it is notable that in this study, there was no significant difference in overall complication rates or in Clavien–Dindo grade III/IV complication rates between patients who received NAC versus those who underwent IS. The Prodige 7 trial, in which the majority of patients had received NAC, demonstrated a relatively low postoperative mortality (1.5%) and 60-day grade III-IV morbidity (13.6%–24.1%) [25]. Similarly, a recent trial examining the use of NAC in patients with ovarian cancer undergoing CRS-HIPEC reported relatively low rates of postoperative complications [31].

Although the current study did not find a significant association between NAC and OS on multivariable analysis, the identified factors associated with OS are consistent with the findings of previous studies. PCI score and completeness of cytoreduction are two of the most important, and reproducibly shown, prognostic factors associated with outcomes of patients undergoing CRS ± HIPEC [7,14]. The finding that synchronous disease was also associated with improved OS is interesting and mirrors that of a recent study in which patients with metachronous disease experienced worse RFS [32]. Of note, the current study excluded patients with extraperitoneal disease as the role of systemic therapy is more established in these patients.

The current study should be interpreted within the context of its limitations, namely its retrospective design. Treatment decisions were not made randomly, and unmeasured differences likely exist between the NAC and SF groups. In preliminary analyses (data not shown), propensity score matching led to similar results but significantly reduced the sample size of the study populations. Other statistical techniques for matching with larger sample sizes could address some of the inherent selection biases. In addition, we were only able to include those patients who successfully completed NAC and underwent surgery. Given the importance of curative-intent surgery on long-term survival, an intention-to-treat analysis may have led to different findings. Finally, given the study’s multi-institutional and retrospective nature, the NAC regimens, number of courses, surgical procedures, and receipt of postoperative therapies were not standardized.

In conclusion, in this multi-institutional retrospective review of patients with CR-PM undergoing CRS ± HIPEC, patients who received NAC experienced a longer OS compared to those who underwent SF, although NAC was not independently associated with improved RFS or OS after controlling for confounding factors. In light of the recent PRODIGE 7 trial in which the majority of patients received perioperative chemotherapy as well as the current study’s findings, NAC followed by CRS with or without HIPEC appears to be a safe and feasible strategy for CR-PM, aiding in the appropriate selection of patients for aggressive cytoreductive surgery. The development of novel therapies that can successfully downstage CR-PM and facilitate complete cytoreduction is needed.

## Figures and Tables

**Table 1:** Patient Characteristics (*N* = 298).

	All Patients		NAC		SF		*p*-Value *
	*n*	%	*n*	(%)	*n*	(%)	
***N***	298	100%	196	66%	102	34%	
**Follow-up (months), mean ± SD (range)**	18.6 ± 18.7 (0.2–124)		19.2 ± 20.1 (0.3–124)		17.3 ± 16.1 (0.2–70.8)		0.372
**Age (years), mean ± SD (range)**	54.1 ± 12.9 (20–95)		53.2 ± 12.5 (22–78)		55.8 ± 13.7 (20–95)		0.121
**BMI**	27.7 ± 6.0 (17.3–60.7)		27.3 ± 6.2 (17.3–60.7)		28.6 ± 5.6 (18.9–43.8)		0.084
**Female**	161	54%	104	53%	57	56%	0.643
**ASA Class**							0.065
I	0	0%	0	0%	0	0%	
II	31	11%	17	10%	14	14%	
III	223	81%	150	85%	73	74%	
IV	22	8%	10	6%	12	2%	
**ECOG Performance Status**							0.432
0	140	59%	83	57%	57	63%	
1	84	36%	54	37%	30	33%	
2	11	5%	8	3%	3	3%	
3	1	0%	8	3%	3	3%	
**Comorbidities**							
Hypertension	100	36%	59	59%	41	41%	0.178
Diabetes	23	8%	17	8%	9	9%	0.729
CHF	8	3%	7	4%	1	1%	0.163
Prior cardiac event	16	6%	9	5%	7	7%	0.496
Prior CVA	5	2%	4	2%	1	1%	0.499
COPD	3	1%	0	0%	3	3%	0.020
CKD	5	2%	4	2%	1	1%	0.683
PVD	13	5%	13	7%	0	0%	0.007
Ascities	20	7%	11	6%	9	9%	0.374
Systemic Anticoagulation	21	7%	17	9%	4	4%	0.128
Chronic Steroid Use	5	2%	5	3%	0	0%	0.092
Rheumatic disease	6	2%	4	2%	2	2%	0.963
**Tobacco use**							0.550
Current	23	8%	17	10%	6	6%	
Former	68	24%	44	25%	24	24%	
**Symptomatic**							
GI Bleed	15	5%	10	5%	5	5%	0.933
Obstruction	15	5%	11	6%	4	4%	0.515
Diarrhea	6	2%	3	2%	3	3%	0.464
Constipation	14	5%	11	6%	3	3%	0.281
Pain	73	26%	48	27%	25	25%	0.706
Nausea/Vomiting	11	4%	8	3%	3	1%	0.541
Anorexia	8	3%	7	4%	1	1%	0.161
Fatigue	29	10%	18	10%	11	11%	0.792
Anemia	37	14%	21	12%	16	17%	0.246
GERD/Dyspepsia	40	15%	35	20%	5	5%	0.001
**Synchronous peritoneal disease**	107	36%	73	37%	34	33%	0.196
**Previous cytoreduction**	49	16%	29	15%	30	20%	0.288
**Previous HIPEC**	12	4%	3	1%	9	3%	0.002
**Tumor Differentiation—Poor and/or Signet Ring Cell**	77	26%	60	31%	17	18%	0.087

* *p*-Values are for comparison of NAC and SF group. NAC, Neoadjuvant Chemotherapy; SF, Surgery First; BMI, Body Mass Index; ASA, American Society of Anesthesiology; ECOG, Eastern Cooperative Oncology Group; CHF, Congestive Heart Failure; CVA, Cerebrovascular Accident; COPD, Chronic Obstructive Pulmonary Disease; CKD, Chronic Kidney Disease; PVD, Peripheral Vascular Disease; GI, Gastrointestinal; GERD, Gastroesophageal Reflux Disease; HIPEC, hyperthermic intraperitoneal chemotherapy.

**Table 2:** Neoadjuvant Chemotherapy Regimens.

	n	%
FOLFIRI + Bevacizumab	45	23.0%
FOLFOX + Bevacizumab	42	21.4%
FOLFOX	37	18.9%
FOLFIRI	20	10.2%
Xeloda + Bevacizumab	9	4.6%
FOLFOXIRI + Bevacizumab	5	2.6%
5FU ± Leucovorin	3	1.5%
Xeloda	3	1.5%
XELOX	3	1.5%
XELOX + Bevacizumab	2	1.0%
Other	24	12.3%
Other + Bevacizumab	3	1.5%

**Table 3:** Tumor and Operative Characteristics.

	All Patients		NAC		SF		*p*-Value *
	*n*	%	*n*	%	*n*	%	
**PCI, mean ± SD (range)**	12.9 ± 8.2 (1–39)		12.1 ± 7.9 (1–38)		14.3 ± 8.5 (2–39)		0.034
CCR							0.218
0	212	71.10%	145	74.00%	67	65.70%	
1	49	16.40%	31	15.80%	18	17.60%	
≥ 2	37	12.40%	20	10.20%	17	16.70%	
HIPEC	281	95.90%	190	96.90%	91	93.80%	0.204
Chemotherapy Mitomycin C	273	97.00%	184	97.40%	89	97.80%	0.822
Oxaliplatin	7	2.50%	5	2.60%	2	2.20%	
HIPEC Infusion Time (minutes), mean ± SD (range)	88.0 ± 9.7 (30–120)		88.4 ± 9.1 (30–120)		87.2 ± 10.7 (30–100)		0.364
Operative Time (hours), mean ± SD (range)	7.8 ± 2.9 (0–20.8)		7.7 ± 2.9 (0–20.8)		8.7 ± 2.7 (2.5–14.5)		0.013
EBL (mL), mean ± SD (range)	437.4 ± 641.5 (0–6000)		447.0 ± 740.9 (0–6000)		419.1 ± 388.9 (0–2000)		0.672
Any postoperative complication	180	60.40%	119	60.70%	61	59.80%	0.879
Highest Clavien–Dindo grade							0.650
I	20	10.90%	11	9.00%	9	14.50%	
II	98	53.30%	64	52.50%	34	54.80%	
III	46	25.00%	34	27.90%	12	19.40%	
IV	15	8.20%	10	8.20%	5	8.10%	
V	5	2.70%	3	2.50%	2	3.20%	
Hospital LOS (days), mean ± SD (range)	12.1 ± 8.0 (0–68)		12.2 ± 8.2 (3–68)		12.1 ± 7.7 (0–49)		0.974
Adjuvant chemotherapy	78	38.60%	62	40.30%	16	33.30%	0.389
Neoadjuvant Radiation	8	3.30%	8	4.30%	0	0%	0.110
Adjuvant Radiation	7	3.80%	5	3.50%	2	4.90%	0.696

* *p*-Values are for comparison of NAC and SF groups. NAC, Neoadjuvant Chemotherapy; SF, Surgery First; PCI, peritoneal carcinomatosis index; CCR, Completeness of Reduction Score; HIPEC, Hyperthermic Intraperitoneal Chemotherapy; EBL, Estimated Blood Loss; LOS, Length of Stay.

**Table 4:** Univariate and Multivariable Cox Regression Analysis for Overall Survival.

	Univariate		Multivariable	
	HR (95% CI)	*p*-Value	HR (95% CI)	*p*-Value
Gender				
Female	Ref	Ref		
Male	1.19 (0.83, 1.70)	0.344		
Age (years)	1.00 (0.98, 1.01)	0.748		
BMI	0.97 (0.94, 1.01)	0.104		
ASA Class				
II	Ref	Ref		
III	1.52 (0.79, 2.94)	0.209		
IV	1.36 (0.58, 3.22)	0.478		
Previous HIPEC				
No	Ref	Ref	Ref	Ref
Yes	1.95 (0.91, 4.19)	0.088	1.46 (0.62, 3.43)	0.384
Symptomatic				
No	Ref	Ref		
Yes	1.30 (0.91, 1.87)	0.143		
Synchronous Peritoneal Disease				
No	Ref	Ref	Ref	Ref
Yes	0.65 (0.45, 0.961)	0.031	0.63 (0.42, 0.94)	0.024
PCI Score	1.07 (1.05, 1.10)	< 0.001	1.05 (1.03, 1.08)	< 0.001
CCR				
0	Ref	Ref	Ref	Ref
1	3.56 (2.29, 5.52)	< 0.001	2.18 (1.31, 3.63)	0.003
≥ 2	4.59 (2.78, 7.57)	< 0.001	2.37 (1.21, 4.67)	0.012
HIPEC				
No	Ref	Ref	Ref	Ref
Yes	0.33 (0.16, 0.68)	0.003	0.57 (0.24, 1.36)	0.204
Tumor Differentiation				
Well Differentiated	Ref	Ref		
Moderately Differentiated	1.35 (0.63, 2.87)	0.442		
Poorly Differentiated	1.40 (0.64, 3.06)	0.402		
Not Reported	1.35 (0.63, 2.89)	0.435		
Any postoperative complication				
No	Ref	Ref		
Yes	1.24 (0.86, 1.79)	0.251		
Neoadjuvant Chemotherapy				
No	Ref	Ref	Ref	Ref
Yes	0.69 (0.48, 0.99)	0.045	0.80 (0.54, 1.17)	0.247
Adjuvant Chemotherapy				
No	Ref	Ref		
Yes	1.00 (0.63, 1.58)	0.998		
Neoadjuvant Radiation				
No	Ref	Ref		
Yes	1.65 (0.60, 4.53)	0.331		
Adjuvant Radiation				
No	Ref			
Yes	0.77 (0.27, 2.16)	0.613		

**Table 5:** Univariate and Multivariable Cox Regression Analysis for Recurrence Free Survival.

	Univariate		Multivariable	
	HR (95% CI)	*p*-Value	HR (95% CI)	*p*-Value
Gender				
Female	Ref	Ref		
Male	0.99 (0.73, 1.36)	0.963		
Age (years)	1.00 (0.99, 1.01)	0.943		
BMI	0.99 (0.96, 1.02)	0.990		
ASA Class				
II	Ref	Ref	Ref	Ref
III	1.56 (0.90, 2.73)	0.116	1.60 (0.90, 2.83)	0.107
IV	2.03 (0.99, 4.17)	0.054	1.90 (0.91, 3.98)	0.089
Previous HIPEC				
No	Ref	Ref		
Yes	1.52 (0.75, 3.11)	0.249		
Symptomatic				
No	Ref	Ref	Ref	Ref
Yes	0.72 (0.52, 1.00)	0.041	0.80 (0.56, 1.14)	0.213
Synchronus Peritoneal Disease				
No	Ref	Ref		
Yes	0.90 (0.65, 1.24)	0.507		
PCI Score	1.04 (1.03, 1.07)	< 0.001	1.05 (1.02, 1.07)	< 0.001
CCR				
0	Ref	Ref	Ref	Ref
1	1.52 (1.02, 2.29)	0.041	0.74 (0.45, 1.22)	0.242
≥ 2	0.82 (0.41, 1.61)	0.557	0.57 (0.26, 1.27)	0.172
HIPEC				
No	Ref	Ref		
Yes	0.59 (0.22, 1.61)	0.306		
Tumor Differentiation				
Well Differentiated	Ref	Ref		
Moderately Differentiated	1.13 (0.62, 2.06)	0.701		
Poorly Differentiated	1.26 (0.68, 2.34)	0.457		
Not Reported	0.64 (0.34, 1.21)	0.173		
Any postoperative complication				
No	Ref	Ref	Ref	Ref
Yes	1.51 (1.09, 2.10)	0.014	1.37 (0.74, 1.47)	0.091
Neoadjuvant Chemotherapy				
No	Ref	Ref	Ref	Ref
Yes	0.89 (0.64, 1.22)	0.458	1.04 (0.74, 1.47)	0.834
Adjuvant Chemotherapy				
No	Ref	Ref		
Yes	1.01 (0.70, 1.46)	0.955		
Neoadjuvant Radiation				
No	Ref	Ref		
Yes	2.13 (0.86, 5.27)	0.101		
Adjuvant Radiation				
No	Ref	Ref		
Yes	0.67 (0.27, 1.66)	0.388		
